# Targeted single-cell proteomic analysis identifies new liquid biopsy biomarkers associated with multiple myeloma

**DOI:** 10.1038/s41698-023-00446-0

**Published:** 2023-09-18

**Authors:** Sonia M. Setayesh, Libere J. Ndacayisaba, Kate E. Rappard, Valerie Hennes, Luz Yurany Moreno Rueda, Guilin Tang, Pei Lin, Robert Z. Orlowski, David E. Symer, Elisabet E. Manasanch, Stephanie N. Shishido, Peter Kuhn

**Affiliations:** 1 Convergent Science Institute in Cancer, Michelson Center for Convergent Bioscience, University of Southern California, Los Angeles, CA 90089 USA; 2 Department of Lymphoma and Myeloma, Division of Cancer Medicine, University of Texas MD Anderson Cancer Center, Houston, TX 77030 USA; 3 Department of Hematopathology, University of Texas MD Anderson Cancer Center, Houston, TX 77030 USA; 4 Catherine & Joseph Aresty Department of Urology, Institute of Urology, Keck School of Medicine, University of Southern California, Los Angeles, CA 90033 USA; 5 Norris Comprehensive Cancer Center, Keck School of Medicine, University of Southern California, Los Angeles, CA 90033 USA; 6 Department of Biomedical Engineering, Viterbi School of Engineering, University of Southern California, Los Angeles, CA 90089 USA; 7 Department of Aerospace and Mechanical Engineering, Viterbi School of Engineering, University of Southern California, Los Angeles, CA 90089 USA; 8 Department of Biological Sciences, Dornsife College of Letters, Arts, and Sciences, University of Southern California, Los Angeles, CA 90089 USA

**Keywords:** Myeloma, Diagnostic markers

## Abstract

Multiple myeloma (MM) is accompanied by alterations to the normal plasma cell (PC) proteome, leading to changes to the tumor microenvironment and disease progression. There is a great need for understanding the consequences that lead to MM progression and for the discovery of new biomarkers that can aid clinical diagnostics and serve as targets for therapeutics. This study demonstrates the applicability of utilizing the single-cell high-definition liquid biopsy assay (HDSCA) and imaging mass cytometry to characterize the proteomic profile of myeloma. In our study, we analyzed ~87,000 cells from seven patient samples (bone marrow and peripheral blood) across the myeloma disease spectrum and utilized our multiplexed panel to characterize the expression of clinical markers for PC classification, additional potential therapeutic targets, and the tumor microenvironment cells. Our analysis showed BCMA, ICAM3 (CD50), CD221, and CS1 (SLAMF7) as the most abundantly expressed markers on PCs across all myeloma stages, with BCMA, ICAM3, and CD221 having significantly higher expression levels on disease versus precursor PCs. Additionally, we identify significantly elevated levels of expression for CD74, MUM1, CD229, CD44, IGLL5, Cyclin D1, UBA52, and CD317 on PCs from overt disease conditions compared to those from precursor states.

## Introduction

Multiple myeloma (MM) is the second leading hematologic malignancy accounting for 34,920 new cases and 12,410 deaths annually in the United States alone^1^. Myeloma initiates in the bone marrow (BM) as a result of the clonal expansion of resident plasma cells (PCs) and is preceded by two precursor states, monoclonal gammopathy of undetermined significance (MGUS) and smoldering MM (SMM). These lead to high tumor burden and organ damage and include MM, relapsed/refractory MM (RRMM), and either primary or secondary plasma cell leukemia (PCL)^2–5^. During the past three decades, the global incidence rate for MM has increased by 126%, with mortality increasing by 94%^6^. Therefore, there is an unmet need to understand the pathway to malignant transformation in myeloma and discover novel biomarkers that can aid clinical diagnostics and serve as targets for therapeutics.

Clonal proliferation of PCs in the BM, the root cause of MM, is accompanied by alterations to the genetic and proteomic profile of PCs, marking a shift from normal to abnormal phenotypes^7–10^. Primary genetic events in myelomagenesis include the dysregulation of cyclin D and chromosomal hyperdiploidy^11^, with secondary events such as multiple chromosomal losses and chromosome 1q amplification occurring as the disease progresses^12–15^. Beyond genomics, abnormal PCs express an altered proteomic profile compared to that of normal PCs^7,16,17^. However, there is currently no single protein marker that can diagnose MM, requiring the clinical workup to rely on multiparameter flow cytometry with varying biomarker combinations for immunophenotyping^18–20^. Moreover, the changes to the proteomic landscape of PCs during MM development, progression, treatment response, and disease relapse have not been fully explored. Given the importance of proteins to serve as targetable markers for diagnosis and treatment, there remains an unmet need for the application of technologies that can provide a more comprehensive proteomic profile of PCs in myeloma settings.

Currently, conventional 4-10 color flow cytometry methods are being used to stratify PCs and monitor disease in the BM of MM patients, mainly during diagnosis, disease monitoring or post-therapy^20^. There are several methods for MM flow cytometry, of which EuroFlow is the most notable^21^, utilizing an eight-color assay on two divided tubes from the sample to ensure a sensitivity of 10^-5^. Studies utilizing flow cytometry for monitoring minimal residual disease (MRD) in MM have shown higher applicability compared to their counterpart genomics-based methods (allele-specific oligonucleotide quantitative PCR (ASOqPCR)/next generation sequencing (NGS)) in patients, demonstrating the relevance of proteomic-based approaches in the clinical assessments of MM^22,23^. However, despite the advances in the implementation of these techniques, flow cytometry-based methods pose major limitations in standardization due to varying biomarker panels and manual gating strategies^22^. Additionally, while flow cytometry has the potential for multiplexing markers, the extent of this capability is limited.

Beyond diagnostics, treatment options for MM have expanded rapidly during the past decade, with progress being made in the use of proteasome inhibitors and immunomodulatory drugs^24,25^. However, despite these advances, myeloma remains mostly a chronic disease with most patients experiencing serial relapse^1,24^. Recent discoveries in targeted immunotherapy have shown promise in improving clinical outcomes for some patients, but their utility is limited due to adverse side effects from off-target toxicity, increased therapy resistance, and tumor escape^26–37^. Increased immunosuppression of the tumor microenvironment can also negatively impact tumor progression and targeted therapy response^38–43^. Furthermore, a prominent mechanism that allows for MM cells to escape immunotherapy is through tight interactions of PCs with the bone marrow microenvironment cells^39–42^. To provide enhanced therapy options for myeloma, we therefore need technologies that can identify additional targetable biomarkers on heterogenous myeloma cells and profile the tumor microenvironment landscape.

**Fig. 1:**
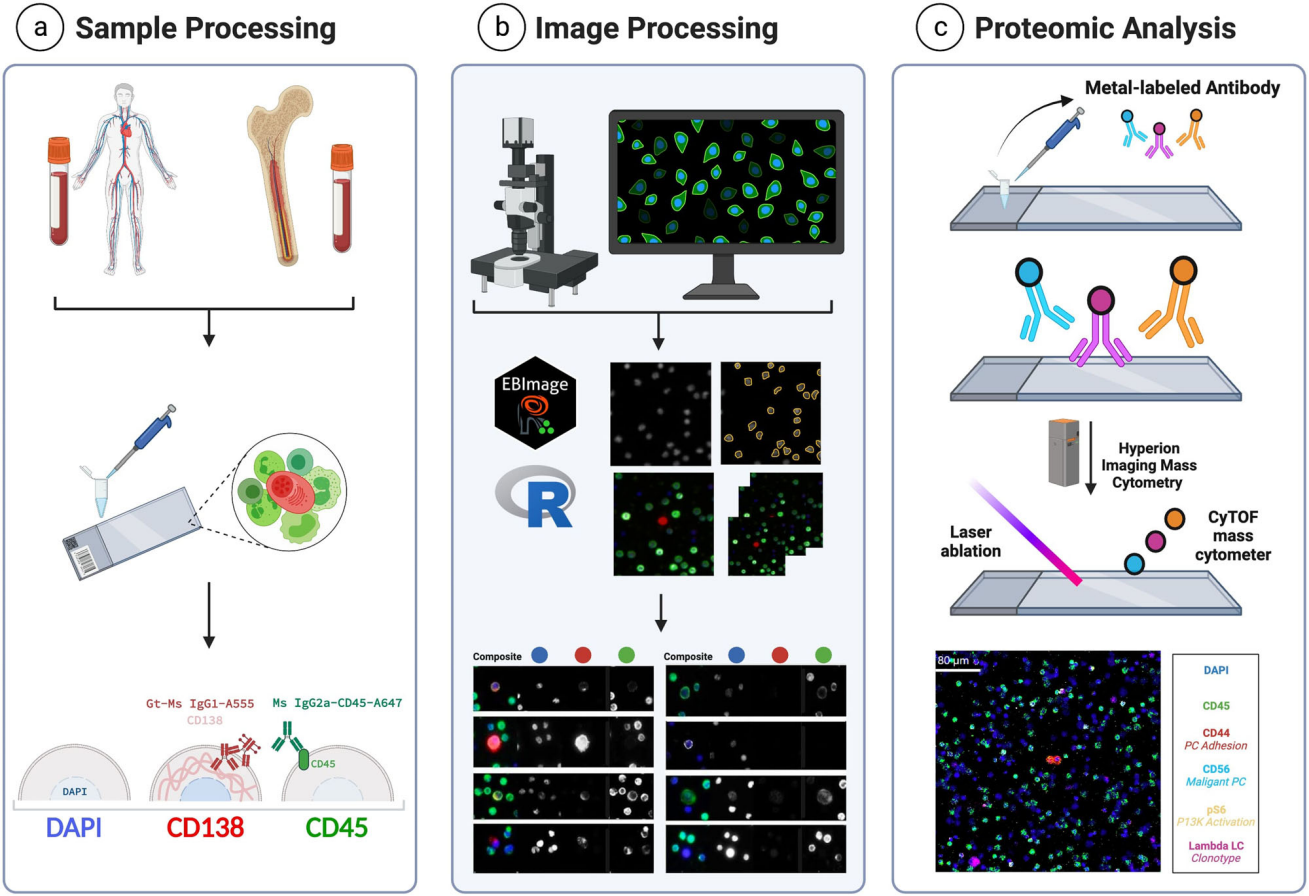
HDSCA-IMC workflow. **a** BMA and PB samples are received, undergo processing by red blood cell lysis, are plated onto custom glass slides at approximately 3 million cells per slide, and are then stained with our 3-color immunofluorescence assay. **b** Stained slides are scanned using high-throughput fluorescence microscopy at 100X magnification, downstream image processing is done via OCULAR, and a final report of cells is generated for HDSCA. **c** Slide is stained with a panel of metal-labeled antibodies, laser ablated using the Hyperion IMC system, and multiplexed images are generated. Created with BioRender.com.

In this study, we demonstrate the applicability of utilizing the high-definition single-cell assay (HDSCA) and imaging mass cytometry (IMC) to identify PCs and further characterize their proteomic expression profiles in a liquid biopsy (Fig. 1). We characterized bone marrow cells in 7 patient samples across the myeloma disease spectrum (2 MGUS, 1 SMM, 2 newly diagnosed MM (NDMM), 1 RRMM, and 1 PCL) and utilized our multiplexed panel to characterize the expression of clinical markers for PC classification, potential therapeutic targets, and the tumor microenvironment cells. Our results from targeted profiling of ~87,000 cells show BCMA, ICAM3, CD221, and CS1 (SLAMF7) as the most abundantly expressed markers on PCs across all myeloma stages, with BCMA, ICAM3, and CD221 having significantly higher expression levels on overt disease conditions, while also being expressed in precursor states. Additionally, we identify significantly elevated levels of expression for CD74, MUM1, CD229, CD44, IGLL5, Cyclin D1, UBA52, and CD317, specifically on PCs from overt disease conditions compared to those from precursor states. Beyond PCs, we were able to further profile the landscape composition of patients’ tumor microenvironment cells.

## Results

### Patient demographics and clinical baseline

In total, this study includes analyses of 7 patients’ samples (6 bone marrow aspirates (BMAs) and 1 peripheral blood (PB)) and 1 normal donor’s PB sample. Patient participants enrolled during 04/2019-03/2020 and were between the ages of 38–72 years at the time of enrollment. Patients’ demographics are provided in Table 1. The sample set included a total of eight slides containing an average of (mean ± standard deviation = 2,339,353.7 ± 209,860.6) nucleated cells for the BMA and an average of (2,443,256 ± 213,971.9) nucleated cells for the PB slides.

**Table 1:** Patient clinical characteristics.

Clinical characteristics	Patient 1	Patient 2	Patient 3	Patient 4	Patient 5	Patient 6	Patient 7
Biopsy site	Bone marrow	Bone marrow	Bone marrow	Bone marrow	Peripheral blood	Bone marrow	Bone marrow
Age	63	54	50	72	45	70	38
Gender	Female	Male	Male	Female	Male	Female	Female
Diagnosis	NDMM	NDMM	RRMM	SMM	PCL	MGUS	MGUS
Serum monoclonal spike (g/dL)	0.4	2.9	0.3	3.9	3.2	0.5	0.4
Serum immunofixation	Positive	Positive	Positive	Positive	Positive	Positive	Positive
Aberrant plasma cell percentage (*Flow cytometry)	95.2	98.8	99.9	78.9	99.8	0	0

### Identification and morphometric analysis of plasma and non-plasma cells

**Fig. 2:**
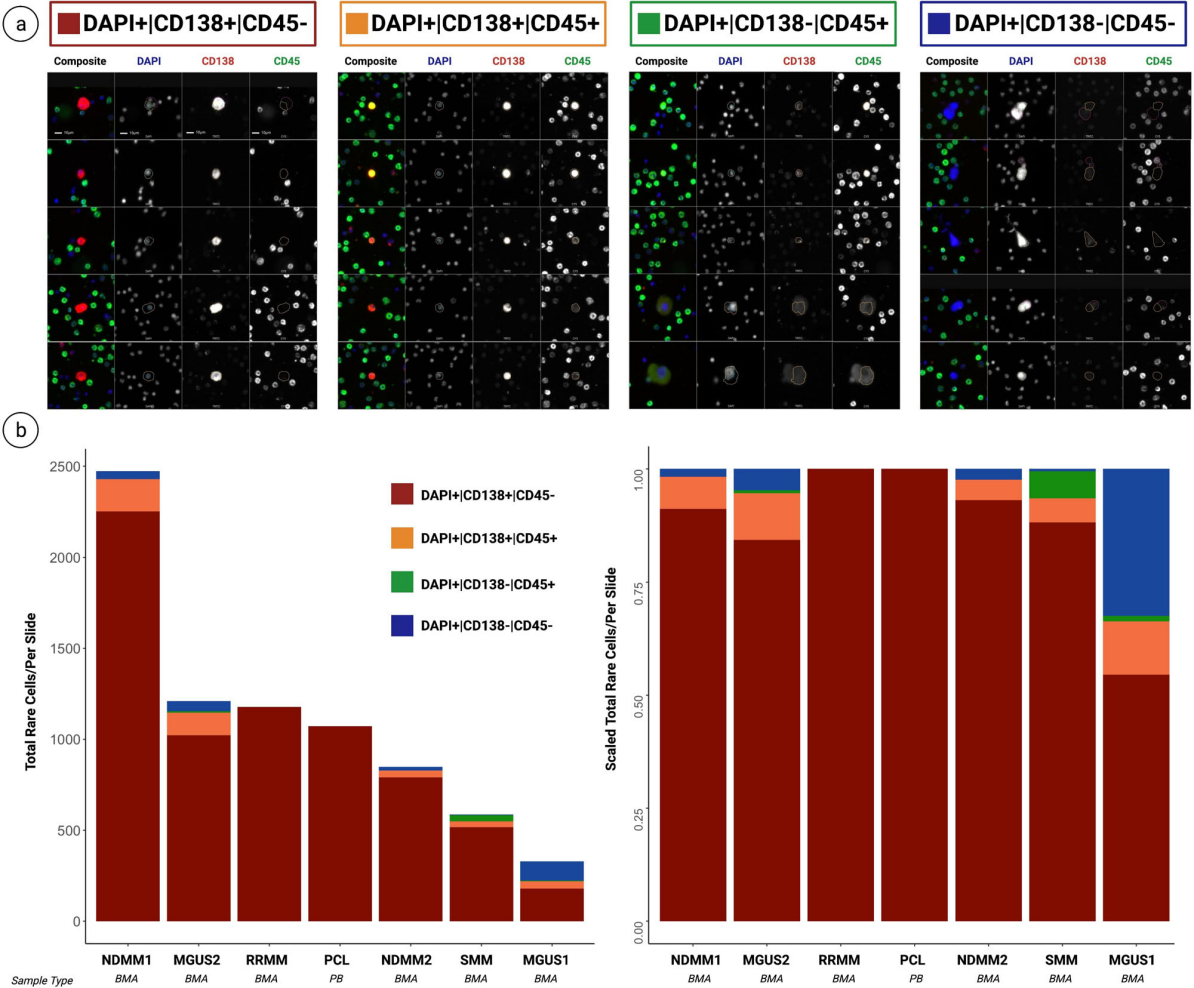
HDSCA3.0 gallery and enumeration of cells. **a** IF representative images of channel classification. Scale bar represents 10 μm. **b** (Left) Enumeration of cell counts per slide (bone marrow for MGUS, SMM, NDMM, and RRMM and peripheral blood for PCL and NBD). (Right) Distribution of total rare cell counts. Scale bar represents 10 μm.

We identified and categorized the candidate cells using an automated rare cell detection workflow followed by manual classification based on three-color immunofluorescence staining, corresponding to DAPI, CD138, and CD45 (Fig. 2a). Additionally, we considered cellular morphology, consisting of cell size and eccentricity, when classifying cells of interest. PCs were identified as DAPI+|CD138+|CD45− and DAPI+|CD138+|CD45+ events and were included in the liquid biopsy profile for all samples. Additional rare events of interest were detected as morphologically distinct DAPI+|CD138−|CD45+ and DAPI+|CD138−|CD45− cells. Enumeration of the events revealed higher total rare cell count in the overt-disease settings of NDMM1, RRMM, PCL, and NDMM2 (mean ± standard deviation = 1,393.25 ± 634.1) compared to precursor states SMM and MGUS1 (mean ± standard deviation = 709.0 ± 370.0) (Fig. 2b).

Our counts identified lower levels of DAPI+|CD138+|CD45− cells in precursor conditions (mean ± standard deviation = 573 ± 346.0), compared to disease settings (mean ± standard deviation =1324 ± 555.4). However, MGUS2 showed elevated levels of DAPI+|CD138+ cells (1022), compared to SMM (517) and MGUS1 (180), providing a profile more similar to patients diagnosed with overt disease. For the DAPI+|CD138+|CD45+ group, the precursor conditions had similar levels of (mean ± standard deviation = 64.67 ± 42.1) cells to the disease settings (mean ± standard deviation = 53.50 ± 71.9) (Fig. 2b).

For the CD138− groups, precursor settings had an incidence of rare DAPI+|CD138−|CD45+ cells (mean ± standard deviation = 15.67 ± 13.8), whereas overt-disease conditions had none. An overall higher number of cells was also seen in precursor conditions for the DAPI+|CD138−|CD45− group (mean ± standard deviation = 55.67 ± 42.5) compared to overt-disease settings (mean ± standard deviation = 15.75 ± 17.7) (Fig. 2b).

The normal control report identified 2 DAPI+|CD138+|CD45− cells and 1 rare DAPI+|CD138−|CD45+ cell, making it the sample with the least number of rare events. Since some of our disease groups studied only had 1 patient sample, we did not perform statistical tests between the groups.

### Multiplexed proteomic profiling of PCs

**Fig. 3:**
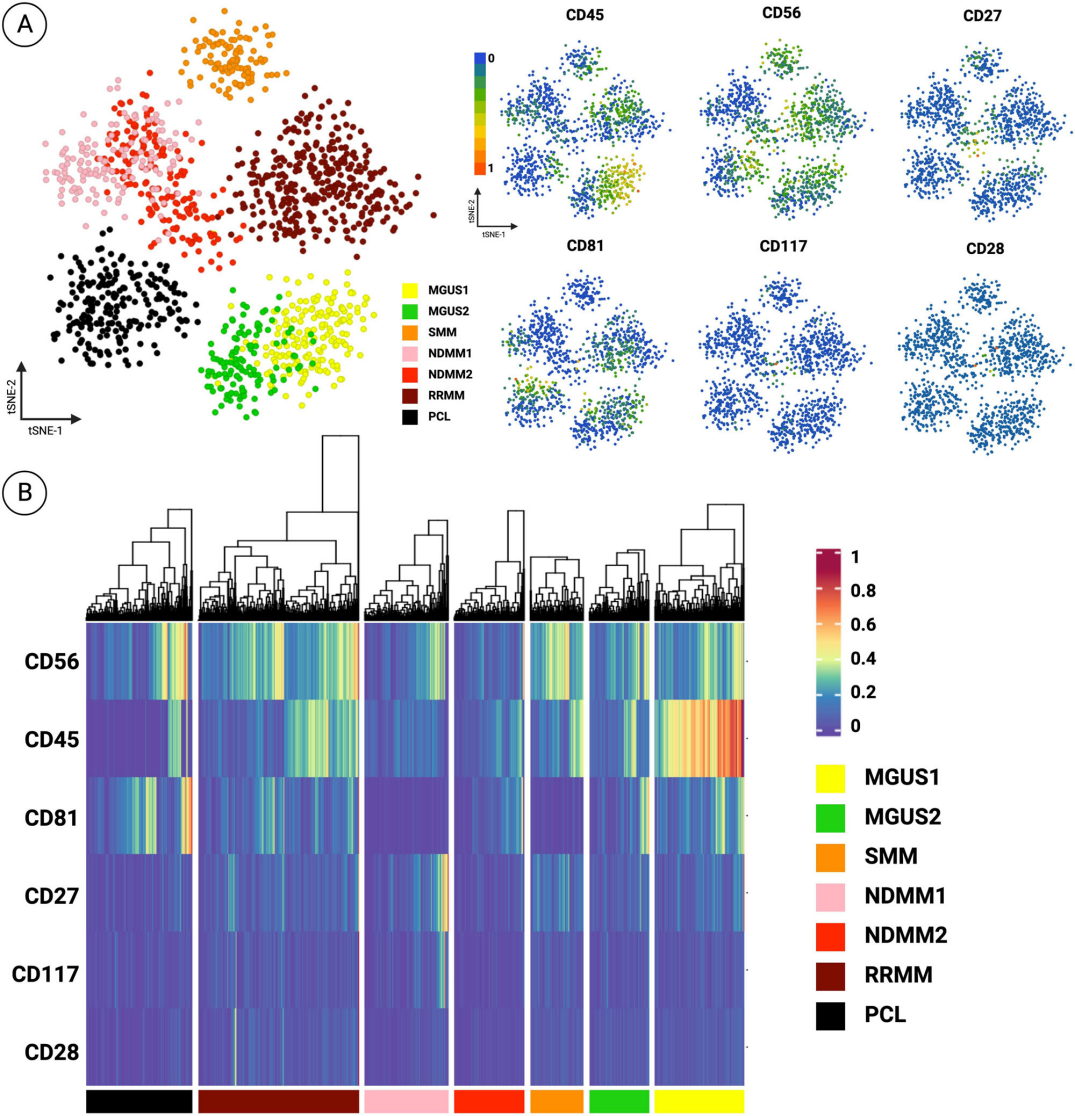
IMC proteomic immunophenotyping of PCs. **A** t-SNE scatter plot of PCs from samples, visualized using only clinical markers (CD45, CD56, CD27, CD81, CD117, CD28), based on 0–1 normalized values. On the left, t-sne is colored by patient ID and on the right, colored by expression levels of biomarkers. **B** Heatmap illustration of clinical markers on patient samples, based on 0–1 normalized values.

Selected candidate PCs (DAPI+|CD138+|CD45+ and DAPI+|CD138+|CD45− cells) were included in each region of interest (ROI) alongside ~300 surrounding white blood cells (WBCs) and were subjected to downstream proteomics. Background WBCs (CD45+CD138-CD38- cells) from slides were used as controls, and the expression levels of markers were normalized on a scale of 0–1. PCs were defined as CD138+CD38+ cells to match with the current clinical definition and flow cytometry gating strategy. We then assessed the normalized expression levels of available clinical biomarkers CD81, CD117, CD56, CD27, and CD28 (Fig. 3).

CD56 was most abundantly expressed on all PCs throughout the disease spectrum, with the highest level of expression in RRMM and PCL samples. CD45 was highest in MGUS settings, with MGUS1 having the most CD45 enriched cells compared to all patients. MGUS2 had lower CD45 levels compared to MGUS1, in line with the clinical observation of its classification as being CD45 Low/Negative (Fig. 3). Our observations of the clinical marker signals show concordance with the clinical classification, although the precise degree of concordance could not be determined as the exact expression levels from clinical flow cytometry were not available (Supplementary Table 2). Additionally, for the IMC and the clinical flow cytometry, two distinct samples collected at the same time were used for assessment, and the antibody clones may differ between the assays.

**Fig. 4:**
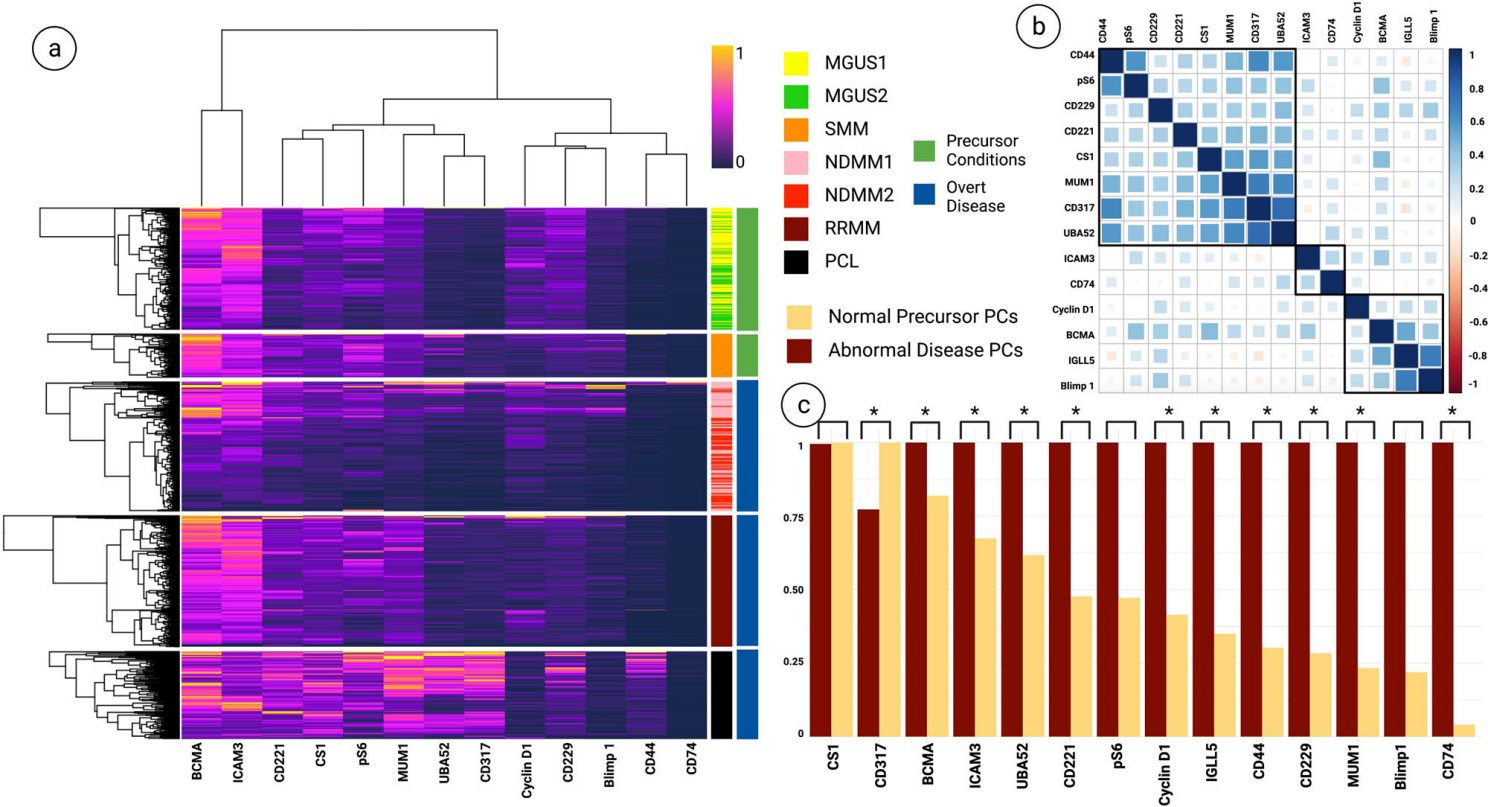
Multiplexed proteomic profiling of PCs. **a** Heatmap representation of CD138+CD38+ PC expression levels for the 14 selected biomarkers (refer to Table 2). Expression levels are depicted using values normalized to a 0–1 scale, which standardizes the different biomarkers for comparison by transforming their expression levels to a common scale. **b** Correlation matrix of CD138+CD38+ PCs from all samples for the selected 14 proteomic markers. Pearson’s correlation was used, and hierarchical cluster analysis was conducted. **c** Bar plot comparing the selected proteomic markers between precursor PCs (from MGUS samples) and disease PCs (from NDMM/RRMM/PCL samples). The Kruskal–Wallis H test (one-way ANOVA) was performed, and *p*-values below *0.05 were considered statistically significant.

To further characterize the proteomic profile of PCs, we investigated the expression of additional biomarkers with the potential to act as MM targets (Fig. 4). Our results demonstrate that BCMA, ICAM3, CD221, and CS1 (SLAMF7) have elevated levels throughout the disease spectrum (Fig. 4a).

To assess the relationships between the selected additional markers, we performed Pearson’s correlation on the expression data on PCs from all samples, followed by hierarchical clustering (Fig. 4b). The results provide 3 distinct cluster sets, with the highest level of overall correlations between CD317/UBA52 (Pearson’s coefficient = 0.78), IGLL5/Blimp1 (Pearson’s coefficient = 0.68), and CD317/MUM1 (Pearson’s coefficient = 0.673). No significant correlation was found between the markers, highlighting the overall heterogeneity of the PCs.

In order to evaluate the potential of the selected markers to serve as MM targets, we set out to compare their expression between normal and abnormal PCs. We pooled PCs from precursor states where no abnormal PCs were detected (<1%) (MGUS1 and MGUS2) and compared their profiles for the selected markers with PCs from patients with NDMM/RRMM/PCL where >95% aberrant PCs were detected by the clinical flow cytometry (Table 1). We performed the Kruskal–Wallis H test (one-way ANOVA), and all *p-*values equal to or below *0.05 were considered statistically significant. Our results indicate that from the most prevalent markers on PCs across the spectrum of disease, BCMA, ICAM3, and CD221 are significantly expressed at higher levels on PCs from NDMM/RRMM/PCL, compared to those from precursor states (*p* = 0.05, *p* < 0.001, *p* < 0.001, respectively) (Fig. 4c).

We also observed significantly higher levels of expression for CD74 (*p* = 0.014), MUM1 (*p* < 0.001), CD229 (*p* < 0.001), CD44 (*p* < 0.001), IGLL5 (*p* < 0.001), Cyclin D1 (*p* < 0.001), UBA52 (*p* < 0.001), and CD317 (*p* < 0.001) on PCs from overt disease conditions compared to those from precursor states (Fig. 4c). Blimp1 and pS6 were also observed to have higher levels of expression on PCs from overt disease versus precursor conditions, however the differences did not reach statistical significance (*p* = 0.671 and *p* = 0.590, respectively) (Fig. 4c).

### Characterizing the bone marrow microenvironment

**Fig. 5:**
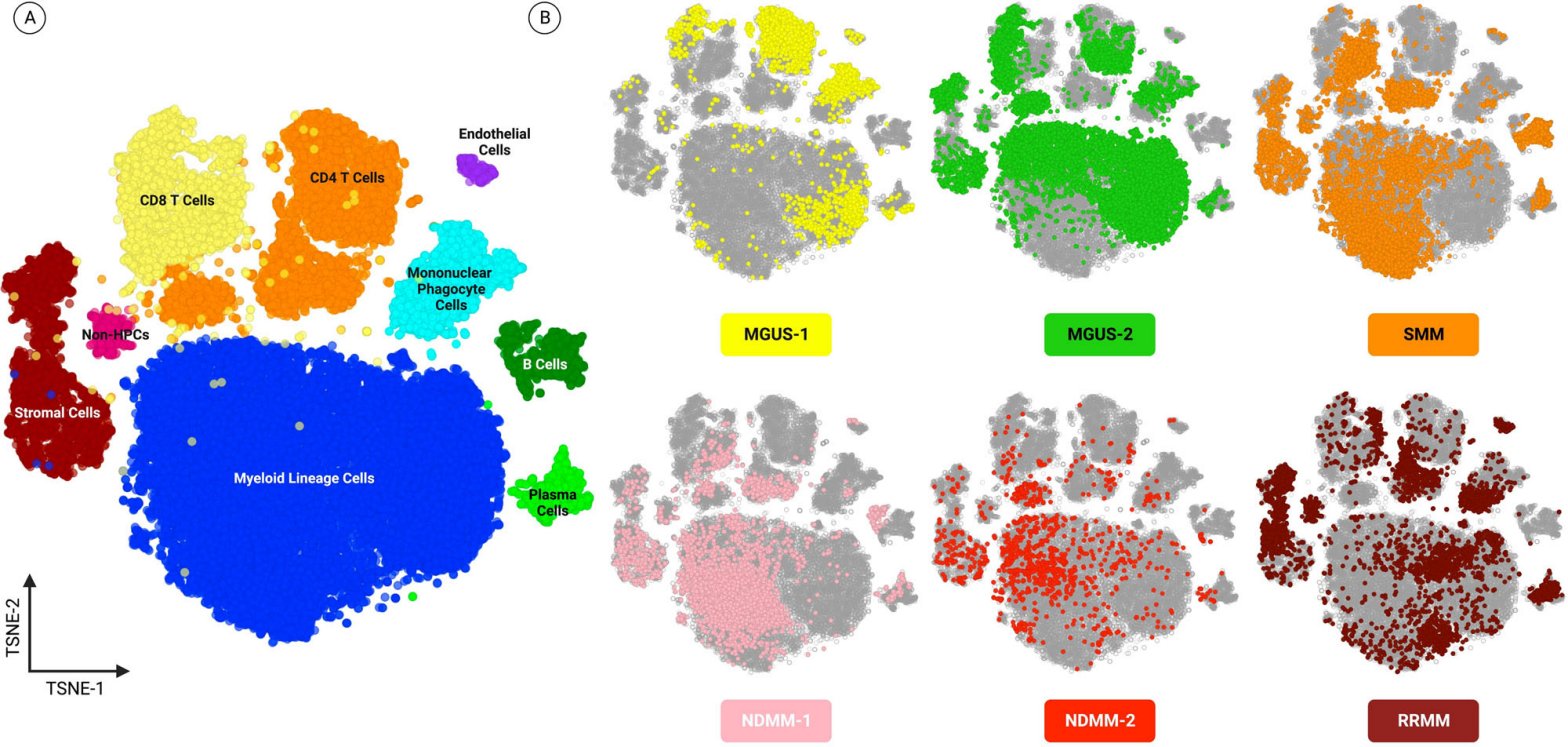
Bone marrow tumor microenvironment landscape across MM stages. Representative cells from the BM samples are shown and profiled based on enriched biomarker expression. **A** Global profile of all samples combined, with clusters colored by assigned cell type. **B** Highlights cells pertaining to each patient sample, with colors assigned to each patient sample, as indicated in the image.

The BMA samples from patients diagnosed with MGUS, SMM, NDMM, and RRMM were further analyzed to profile the tumor microenvironment landscape. Representative cells from each BMA sample were profiled based on their marker expression and cell types were determined using multiplexed quantitative proteomic signals (Supplementary Table 3; Fig. 5).

The bone marrow microenvironment analysis showed an overall significantly lower percentage of T cells detected in the BMA of overt disease conditions (NDMM1 = 7.24%/NDMM2 = 10.60%/RRMM = 7.78%; Overt disease = 8.57 ± 1.45), compared to precursor states (MGUS1 = 16.87%/MGUS2 = 14.62%/SMM = 17.0%; Precursor = 16.16 ± 1.09, *p* = 0.003; Fig. 5).

## Discussion

Here, we demonstrate the applicability of utilizing the single-cell high-definition liquid biopsy assay (HDSCA) and imaging mass cytometry (IMC) to identify PCs and further characterize their proteomic expression profiles. By utilizing a panel of antibodies that correspond to current clinical markers used for myeloma cell immunophenotyping, additional biomarkers for targeted therapy, and immune markers, we are able to characterize the proteomic profile of disease for clinical classification, evaluate biomarkers for targeted therapy, and provide an overview of the immune landscape all in one assay.

Our analysis showed upregulated expression of BCMA, ICAM3, CD221, and CS1 (SLAMF7) in patients across all disease states. BCMA, a member of the TNF17 receptor family, has been found to be expressed on late-stage B-cell lymphocytes (memory B cells mainly), normal as well as abnormal plasma cells^44–46^. Targeted BCMA therapies have revolutionized the myeloma treatment landscape with unparalleled efficacy leading to improved clinical outcomes for late relapse patients^32–34,37^. However, there are currently no approved diagnostic tools that specifically measure cellular BCMA levels, limiting our ability to evaluate its expression throughout the course of the disease and routinely monitor the effects of anti-BCMA therapies. Utilizing targeted panels that measure BCMA could improve anti-BCMA therapy response monitoring in such patients. ICAM3, a type I transmembrane glycoprotein, has been previously identified to be upregulated on the surfaceome of MM cell lines and may play a role in immune evasion of natural killer cell-initiated lysis^17,47^. Our results identified ICAM3 as an upregulated biomarker in patients across MM stages, with significantly higher expression in overt disease compared to precursor states supporting its potential significance. Insulin-like growth factor-1 receptor (CD221) has been previously shown to be upregulated on human myeloma cells, with Bataille et al. demonstrating CD221 expression level as a negative prognostic marker for MM patients^48^. CS1 (SLAMF7) has demonstrated uniformly high expression on MM cells, regardless of genomic heterogeneity and disease state^49,50^. Importantly, Elotuzumab, a humanized anti-CS1 (SLMAF7), is approved for the treatment of relapsed myeloma with new target products in development^51^. Our analysis confirms both CD221 and CS1 (SLAMF7) to be highly expressed in myeloma cells, in agreement with previous studies.

Furthermore, we were also able to identify additional biomarkers that were more specifically expressed in overt disease conditions compared to precursor states. Markers such as CD74, MUM1, CD229, CD44, IGLL5, Cyclin D1, UBA52, and CD317 have the potential to act as emerging biomarkers for targeted therapies, pending future studies that can demonstrate their utility on a larger scale.

The core limitation of our study was the low number of patients we were able to include and not being able to provide a longitudinal study of each patient. Additionally, for the PCL patient, we were not able to receive a BMA sample, as the clinical workup was limited to a blood draw. In the future, we hope to recruit a higher number of patients and obtain matched PB and BMA samples across the MM spectrum to better characterize the disease. Validation in a larger cohort of MM patients can further help to understand the potential utility of our identified proteins as early diagnostic and prognostic markers.

Our goal was to demonstrate the utility of the HDSCA and IMC to provide a comprehensive profile of MM disease. MM is primarily diagnosed in the advanced stages, and currently, the medical interventions for treatment are limited. Early diagnosis and identification of additional therapeutic targets of MM are crucial as they provide chances for better disease management and can improve patient care and survival. We believe that a multiplexed proteomic panel could assist in future MM disease management, providing alternative therapeutic targets that have the potential to improve the survival expectancy of patients. Identifying such protein-based biosignature of MM from the liquid biopsy has the potential to serve as an early predictor of disease development and progression.

## Methods

### Patient enrollment

A total of 7 patients and one normal blood donor (ND) are included in this study. All patients, except the ND, were recruited at The University of Texas MD Anderson Cancer Center (Houston, TX) (IRB: UP-19-0033) between 2019 and 2020 (Table 1). For diagnosis purposes, all participating patients received a bone marrow biopsy and serological testing. A corresponding sample from each patient underwent standard-of-care flow cytometry analysis by MD Anderson as part of the MM diagnostic workup. At the time of sample collection, 2 patients were diagnosed with MGUS, 1 with SMM, 2 with NDMM, 1 with RRMM, and 1 with PCL (Table 1). The NBD sample was acquired from the Scripps Clinic Normal Blood Donor Service from an individual with no known pathology. Bone marrow aspirates (BMA) were collected from MGUS, SMM, NDMM, and RRMM patients, while peripheral blood (PB) was collected for PCL and ND. Recruitment took place according to institutional review board-approved protocols at MD Anderson Cancer Center, and all study participants provided written informed consent.

### Sample collection and processing

All PB and BMA samples (8 mL) were collected in Streck tubes (Cell-free DNA Blood Collection Tube, Streck, La Vista, NE, USA) at MD Anderson and shipped to the Convergent Science Institute in Cancer at University of Southern California within a 48-h time period, as previously described^52,53^. Immediately upon receipt, all samples underwent red blood cell lysis in isotonic ammonium chloride solution, and the remaining nucleated cell population was plated in a monolayer on custom-made cell adhesive glass slides (Marienfeld, Lauda, Germany). The WBC count of the sample was used to approximate plating 3 million cells per slide. The prepped slides were subsequently incubated in 7% BSA, dried, and stored at −80 °C^52–54^.

### Immunostaining and image acquisition

Slides were thawed prior to immunofluorescent staining. All steps were performed at room temperature using an IntelliPATH FLX™ autostainer (Biocare Medical LLC, Irvine, CA, USA). Slides received 2% neutral buffered paraformaldehyde solution (VWR, San Dimas, CA) for 20 min for cell fixation and were subsequently incubated with 10% goat serum (Millipore, Billerica, MA) for 20 min to block non-specific binding sites. The slides were then stained with a primary antibody cocktail containing mouse anti-human CD138 (B-A38, MCA2459GA, Bio-Rad, Hercules, CA) and mouse anti-human CD45 Alexa Fluor® 647 monoclonal antibody (F10-89-4, MCA87A647, AbD Serotec, Raleigh, NC), for 1 h. Antibodies had previously been validated as a part of the assay development study for MM^55^. The slides were washed with TBS after primary staining. Thereafter, slides were incubated with goat anti-mouse Alexa Fluor® 555 (A21127, Invitrogen, Carlsbad, CA) and counterstained with 4, 4-diamidino-2-phenylindole (DAPI; D1306, Thermo Fisher, Waltham, MA) for 40 min. Finally, all the slides were mounted with a glycerol-based media, cover-slipped and sealed for subsequent imaging^52,54,55^.

The immunofluorescent-stained slides were then imaged using automated high-throughput fluorescence scanning microscopy at 100X magnification, which was used to obtain 2304 frame images per fluorescence channel per side, as previously described^54^. Background noise levels on all slides, the gain and exposure times for all channels, DAPI (DNA), Alexa647 (CD45), and AlexaFluor®555 (CD138) were standardized for background normalization by the scanner software. After images were captured, 2304 frames per slide, cells were segmented and had their features extracted via customized EBImage^56^ and R software (R version 4.1.2, R core team, 2021)^57^.

### Cell classification and region of interest identification

We utilized a customized algorithm known as OCULAR (Outlier Clustering Unsupervised Learning Automated Report) to identify and categorize rare cells in our study. This innovative image-processing pipeline combines dimensionality reduction, segmentation, and unsupervised clustering methodologies, using principal component analysis and unsupervised learning ^55,57,58^.

Initially, OCULAR employs the ‘EBImage’ R package to segregate DAPI-positive cells and DAPI-negative events. It then extracts features for each cell, generating a comprehensive array of 761 cellular and nuclear parameters. Utilizing principal component analysis (PCA) on these parameters and conducting hierarchical clustering based on the top 350 components, OCULAR can identify both common and rare cells from all DAPI+ events. This process is deterministic, ensuring the repeatability and robustness of the results.

OCULAR further enhances its categorization by performing a K-nearest neighbor analysis on the cellular morphological features, which include marker signal intensity and cellular and nuclear shape and size ^55,57,58^. It subsequently categorizes cells into three main groups: (1) Plasma cells, identified using CD138 as the marker (DAPI+|CD138+|CD45− and DAPI+|CD138+|CD45+), (2) DAPI+ only cells (DAPI+|CD138−|CD45−), and (3) non-Plasma Cell hematopoietic cells (DAPI+|CD138−|CD45+). In addition to these, OCULAR identifies ‘morphologically distinct’ cells that possess unique size, shape, and eccentricity compared to surrounding White Blood Cells ^55,57,58^.

For further characterization, an average of 34 regions of interest (ROI) on the slide were selected in the BMA and PB samples to undergo downstream targeted proteomics analysis (Supplementary Table 1). ROIs were selected as regions on the slide that had at least 1 DAPI+|CD138+ cell.

### Targeted proteomics using imaging mass cytometry

For the downstream multiplexed proteomics, we utilized the Hyperion imaging mass cytometry (IMC) system, as previously described^59,60^. Metal-labeled antibodies were validated in the HDSCA workflow to ensure the specificity, selectivity, and reproducibility of antibodies through testing on biomarker-specific cell lines spiked into normal blood donor samples and spread on slides^59^.

The slides were stained with an MM-specific panel that utilized 35 metal-labeled antibodies and 2 DNA intercalators to characterize normal and abnormal PCs and BM microenvironment cells and to further characterize MM (Table 2). All antibodies were prepared at a standard dilution of 1:100. For BCMA, two antibody clones were tagged to the same metal (Nd150). Prior to staining with the metal-labeled antibody cocktail for IMC analysis, the slides were stored at 4 °C for 15-78 days (mean 50.4 ± 25.1). Metal-labeled antibodies were retrieved as either direct conjugates from Fluidigm (now Standard BioTools; San Francisco, CA) or underwent in-house conjugation, as per Maxpar’s antibody labeling protocol instructions (Table 2). The cocktail of metal-labeled antibodies was prepared in 1% BSA and 0.1% Tween in PBS^59^. The slides were removed from the 4 °C refrigerator, had their coverslips taken off, and were dipped in fresh PBS twice to wash off the glycerol-based mounting media prior to staining. Slides were then blocked with a buffer containing 1% BSA and IgG mouse Fc fragments (31205, Thermo Scientific, Waltham, MA) for 1 h and then incubated with the antibody cocktail for 1.5 hr on an orbital shaker at room temperature. Afterward, the slides were washed with fresh PBS and subsequently incubated with DNA intercalators (Ir191/Ir193, 201192A, Fluidigm, San Francisco, CA) for 30 min. Finally, the slides were washed with PBS and ddH_2_O, dried, and stored at room temperature until ablation (approximately for 1–4 days).

**Table 2:** IMC multiplexed proteomic analysis panel of metal-labeled antibodies.

Biomarker	Host+clone	Metal tag	Vendor	Lot#	CAT#
CD20	Rabbit_IgG_SP32	Nd142	Abcam	GR3246631-1	ab236434
CD38	Rabbit_IgG_EPR2269-219	Sm152	Abcam	GR3279334-2	ab255693
CD81	Rabbit_IgG_EPR21916	Er167	Abcam	GR3219804-1	ab233692
CD4	Rabbit_IgG_EPR6855	Yb176	Abcam	GR3215375-18	ab181724
lambda light chain	Rabbit_IgG_EPR5367-62	Eu151	Abcam	GR308254-4	ab185131
CD31	Rabbit_IgG_EPR3094	Er168	Abcam	GR3229164-2	ab207090
CD28	Rabbit_IgG_EPR22076	Eu153	Abcam	GR3252786-6	ab243557
MUM1	Rabbit_IgG_EP5699	Gd155	Abcam	GR3255392-1	ab240071
BCMA	Rabbit_IgG_EPR22457-260	Nd150	Abcam	GR3272148-2	ab254205
BCMA	Rabbit_IgG_EPRBOB-R1-F1-24	Nd150	Abcam	GR3323392-1	ab254206
c-kit/CD117	Rabbit_IgG_YR145	Nd145	Abcam	GR3263196-1	ab216450
SLAMF7/CS1	Rabbit_IgG_EPR22948-114	Sm147	Abcam	GR3285922-2	ab256529
kappa light chain	Rabbit_IgG_EPR5539-105-4	Tm169	Abcam	GR3299698-1	ab248738
Syndecan/CD138	Rabbit_IgG_EPR6454	Nd148	Abcam	GR3243140-3	ab216458
CD27	Rabbit_IgG_EPR8569	Nd144	Abcam	GR3349592-1	ab256583
PRDM1/Blimp1	Rabbit_IgG_EPR16655	Nd146	Abcam	GR3283733-2	ab240344
CD63	Rabbit_IgG_EPR22458-280	Sm154	Abcam	GR3270800-2	ab254011
ICAM3	Rabbit_IgG_EPR3994-123	Gd158	Abcam	GR3321106-1	ab247851
BAFF/CD257	Rabbit_IgG_EPR22238	Gd160	Abcam	GR3258542-1	ab245833
CD74	Rabbit_IgG_EPR4064	Dy161	Abcam	GR3296796-1	ab247655
IGF1 receptor/CD221	Rabbit_IgG_EPR19322	Dy163	Abcam	GR3351890-1	ab232380
BST2/Tetherin/CD317	Rabbit_IgG_EPR20202-169	Dy164	Abcam	GR3252792-1	ab243563
Cyclin D1	Rabbit_IgG_SP4	Er166	Abcam	GR3344254-1	ab239794
UBA52	Rabbit_IgG_EPR4546	Lu175	Abcam	GR3312845-1	ab247799
CD56	Mouse_IgG2b k_NCAM16.2	Sm149	Fluidigm	3171506	3149021B
pS6	Mouse_IgG1_N7-548	Yb172	Fluidigm	2001806	3172008A
CD3	Rabbit_IgG_Polyclonal	Er170	Fluidigm	1631906/1011903	3170019D
CD44	Rat_IgG2bk_IM7	Yb171	Fluidigm	3421608/1201828	3171003B
CD61	Mouse_IgG1_VI-PL2	Bi209	Fluidigm	981514	3209001B
CD45-RO	Mouse_IgG2a_UCHL1	Yb173	Fluidigm	2141813	3173016D
CD45	Mouse_IgG1k_HI30	Y89	Fluidigm	1631909	3089003B
CD8a	Rabbit_IgG_D8A8Y	Dy162	Fluidigm	1631902	3162035D
HLA-DR	Mouse_IgG2ak_L243	Yb174	Fluidigm	1581613	3174001B
IGLL5	Rabbit_IgG_polyclonal	Nd143	Thermo Fisher	VH3053278	PA5-49022
CD229	Rabbit_IgG_polyclonal	Tb159	Thermo Fisher	VH3053451A	PA5-21135
DNA1	Cell-ID™ Intercalator	Ir191	Fluidigm	201192A	NA
DNA2	Cell-ID™ Intercalator	Ir193	Fluidigm	201192A	NA

*NA* not available.

Laser ablation was conducted on previously identified ROIs across the specimen slides. The protein expression images had a resolution of 1 μm^2^ across the region of interest (400 × 400 μm), allowing for limited characterization of sub-cellular localization. Ion mass data were collected and used for reconstruction of the 1 μm^2^ ROI spatial resolution, 36-dimensional images of the ROI. The number of ROIs run per slide is listed in the supplementary information (Supplementary Table 1).

Cell boundary segmentations and pixel values were determined by a customized pipeline that utilizes CellProfiler^61^, ilastik pixel classifier^62^, and histoCAT^63^. CellProfiler (version 3.18) was used to remove strong outlier pixel signals, “hot pixels”, scale the images at 2x, and prepare image crops for ilastik pixel classifier training. Classification of nuclei, membrane, and background segmentations was done using ilastik (version 1.3.3) and exported as probabilities masks. After visual confirmation of masks, the classifier was applied to the entire dataset using batch processing. After the generation of segmentation masks, files were generated for histoCAT with masks for all ROIs. Single-cell measurements were extracted from all channels using the scikit-image package^64^ in Python 3 programming language (Python Software Foundation, Scotts Valley, CA). We calculated the signal output as the mean ion counts detected over the cell area, with the background signal—determined from the negative mask space—being subtracted from the cell values. We included all cells that demonstrated DNA signals in the final analysis. We then assessed the distribution of the signal for each marker in relation to negative control cell subsets, using background WBCs (defined as CD45+CD138−CD38− cells) from slides as controls. To facilitate comparison across different biomarkers, we normalized the proteomic expression levels for each biomarker to a scale ranging from 0 to 1.

### Data analysis and visualization

Data analysis and visualization were conducted using R (Version 4.1.1., Boston, MA), the Python programming language (Version 3.0, Python Software Foundation, Scotts Valley, CA), and the Orange 3.0 data-mining toolbox in Python^65^. Groups were compared using the Kruskal–Wallis test (a one-way ANOVA on ranks) to detect non-parametric rank-based dependence between multiple groups. This test was used to determine whether the distributions have a median shift greater than the null hypothesis. *p*-values below 0.05 were considered statistically significant. No correction was performed, as the comparisons were planned comparisons. Finally, Pearson’s correlation was used to evaluate the relationship between parameters.

### Reporting summary

Further information on research design is available in the Nature Research Reporting Summary linked to this article.

### Supplementary information

Supplemental Material

Reporting Summary

## Data Availability

All data discussed in this manuscript are included in the main manuscript text and the Supplementary Materials. The images of the single cells are available through the BloodPAC Data Commons Accession ID “BPDC000132” and the permalink https://data.bloodpac.org/discovery/BPDC000132. Additionally, the immunofluorescence image data is available in figshare at and the image mass cytometry data is available in figshare at 10.6084/m9.figshare.23929635.

## Code availability

The code used to analyze the single data uses standard third-party open-source libraries and packages in R and Python. The code used to identify CTCs in the HDSCA workflow uses custom code and is proprietary and licensed to Epic Sciences for commercial use.
