# Supplementary material for: Targeted single-cell proteomic analysis identifies new liquid biopsy biomarkers associated with multiple myeloma
Source: NPJ Precis Oncol. 2023 Sep 18;7:95. doi: 10.1038/s41698-023-00446-0 (PMC10507120; doi:10.1038/s41698-023-00446-0)

**Supplementary Information**

**Supplementary Table 1.** ROI Counts for Slides.


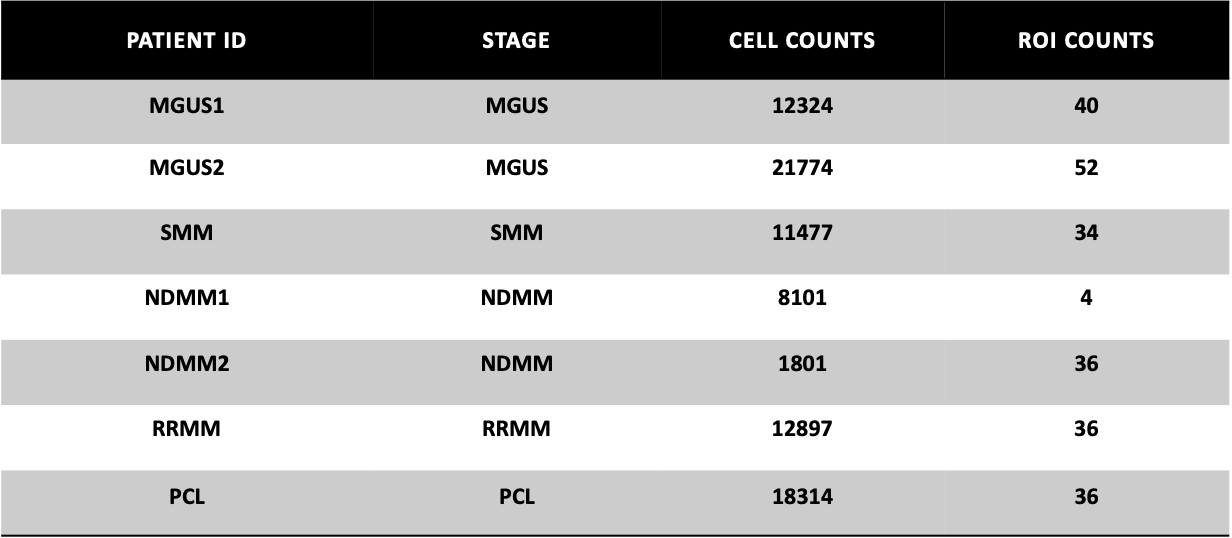


**Supplementary Table 2.** Clinical flow cytometry immunophenotype of PCs.

**
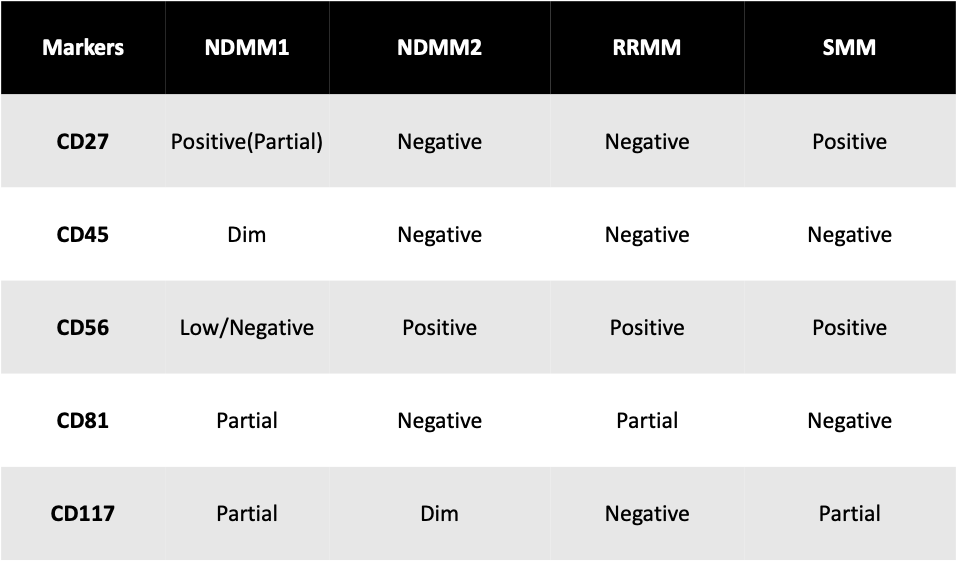
**

**Supplementary Table 3.** Imaging Mass Cytometry Multiplexed Signal**.** Biomarker expression to define each cell subset is provided; Red: "negative" and Black: "positive".


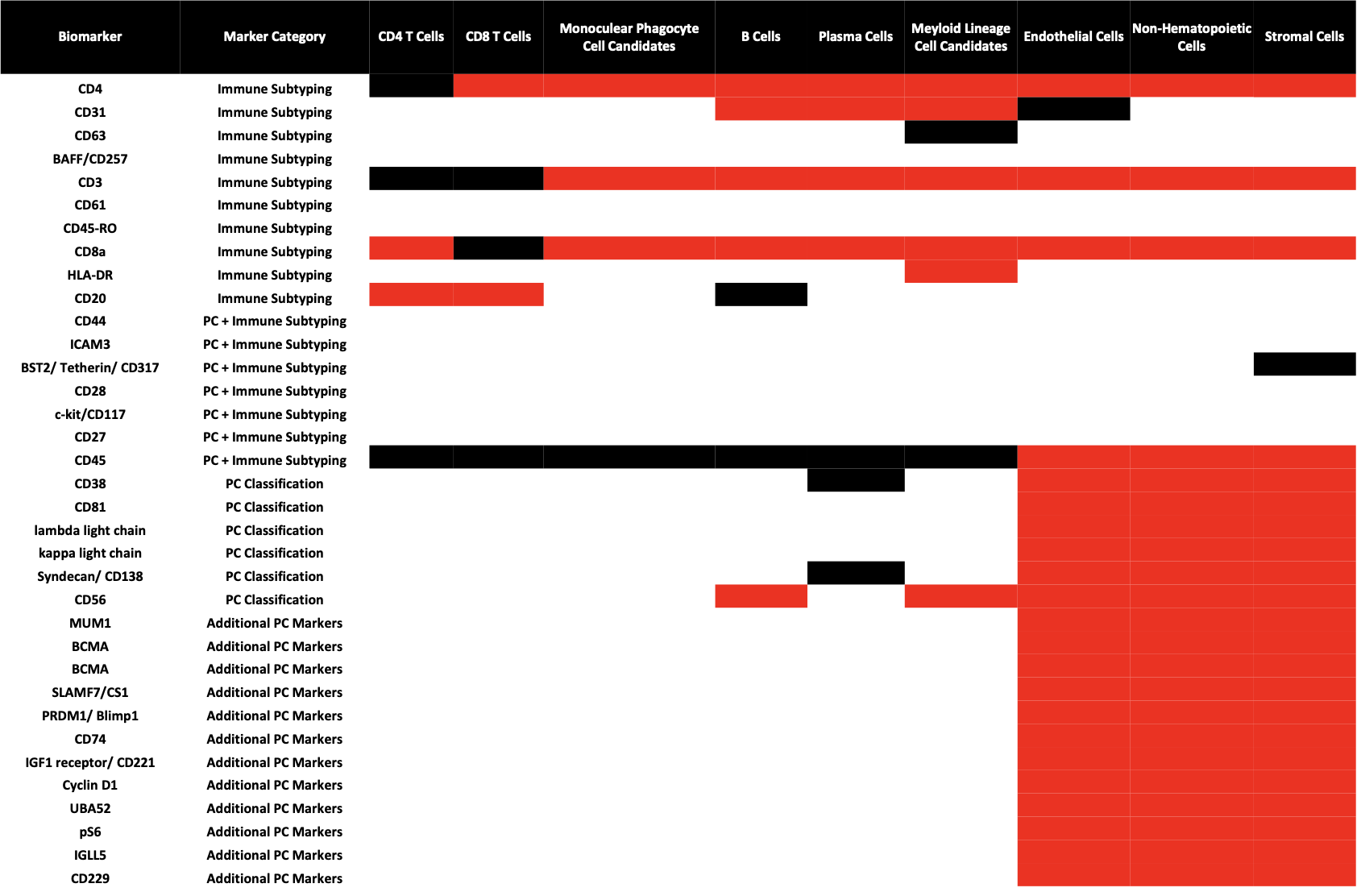

Supplement: Supplementary file 1 — Supplemental Material [file 41698_2023_446_MOESM1_ESM.docx]
