# Supplementary material for: Targeted single-cell proteomic analysis identifies new liquid biopsy biomarkers associated with multiple myeloma
Source: NPJ Precis Oncol. 2023 Sep 18;7:95. doi: 10.1038/s41698-023-00446-0 (PMC10507120; doi:10.1038/s41698-023-00446-0)
Supplement: Supplementary file 2 — Reporting Summary [file 41698_2023_446_MOESM2_ESM.pdf]

Corresponding author(s): Peter Kuhn, PhD

Last updated by author(s): Dec 12, 2022

Reporting Summary

Nature Portfolio wishes to improve the reproducibility of the work that we publish. This form provides structure for consistency and transparency in reporting. For further information on Nature Portfolio policies, see our Editorial Policies and the Editorial Policy Checklist.

Statistics

For all statistical analyses, confirm that the following items are present in the figure legend, table legend, main text, or Methods section.

n/a	Confirmed	
☐	☒	The exact sample size (*n*) for each experimental group/condition, given as a discrete number and unit of measurement
☐	☒	A statement on whether measurements were taken from distinct samples or whether the same sample was measured repeatedly
☐	☒	The statistical test(s) used AND whether they are one- or two-sided<br>*Only common tests should be described solely by name; describe more complex techniques in the Methods section.*
☐	☒	A description of all covariates tested
☐	☒	A description of any assumptions or corrections, such as tests of normality and adjustment for multiple comparisons
☐	☒	A full description of the statistical parameters including central tendency (e.g. means) or other basic estimates (e.g. regression coefficient) AND variation (e.g. standard deviation) or associated estimates of uncertainty (e.g. confidence intervals)
☐	☒	For null hypothesis testing, the test statistic (e.g. *F*, *t*, *r*) with confidence intervals, effect sizes, degrees of freedom and *P* value noted<br>*Give P values as exact values whenever suitable.*
☒	☐	For Bayesian analysis, information on the choice of priors and Markov chain Monte Carlo settings
☒	☐	For hierarchical and complex designs, identification of the appropriate level for tests and full reporting of outcomes
☐	☒	Estimates of effect sizes (e.g. Cohen's *d*, Pearson's *r*), indicating how they were calculated

Our web collection on statistics for biologists contains articles on many of the points above.

Software and code

Policy information about availability of computer code

Data collection	The code used to analyze the single cell data uses standard third-party open-source libraries packaged in R and Python. The code used to identify rare cells in the HDSCA workflow uses custom code and is proprietary and licensed to Epic Sciences for commercial use.
Data analysis	Statistical two-sided analyses were performed using R (Version 4.1.1., Boston, MA). Groups were compared using Kruskal-Wallis (one-way ANOVA on ranks) for non-parametric rank-based dependence between multiple groups to compare whether the distributions have a median shift greater than the null hypothesis. P values below 0.05 were considered statistically significant. No correction was conducted as the comparisons were planned comparisons. Pearson correlation was used to evaluate the relationship between study groups.

For manuscripts utilizing custom algorithms or software that are central to the research but not yet described in published literature, software must be made available to editors and reviewers. We strongly encourage code deposition in a community repository (e.g. GitHub). See the Nature Portfolio guidelines for submitting code & software for further information.

Data

Policy information about availability of data

All manuscripts must include a data availability statement. This statement should provide the following information, where applicable:

- Accession codes, unique identifiers, or web links for publicly available datasets
- A description of any restrictions on data availability
- For clinical datasets or third party data, please ensure that the statement adheres to our policy

Data Availability Statement: All data discussed in this manuscript are either included in the main manuscript text. Some of the data can be accessed through our website.

Human research participants

Policy information about studies involving human research participants and Sex and Gender in Research.

Reporting on sex and gender	Sex and gender were not reported in this study as they were not relevant to our research.
Population characteristics	All participating patients received a bone marrow biopsy, and serological testing. A corresponding sample from each patient underwent standard of care flow cytometry analysis by MD Anderson as part of the MM diagnostic workup. At the time of sample collection, 2 patients were diagnosed with MGUS, 1 with SMM, 2 with NDMM, 1 with RRMM, and 1 with PCL (Table 1). The NBD sample was acquired from the Scripps Clinic Normal Blood Donor Service from an individual with no known pathology. Patients were between the ages of 38-72 yrs at the time of enrollment
Recruitment	Patient participants enrolled during 4/2019-03/2020 and recruitment took place at MD Anderson Cancer Center, with approval from institutional board review and patients providing informed consent.
Ethics oversight	This study was conducted according to the guidelines of the Declaration of Helsinki and approved by the Institutional Review Board (or Ethics Committee) of the University of Southern California's Keck School of Medicine (protocol HS-19-0033 approved on 7 August 2019) and the Anderson Cancer Center (protocol PA18-1073 approved on 14 September 2013 and last approved on 24 March 2021).

Note that full information on the approval of the study protocol must also be provided in the manuscript.

Field-specific reporting

Please select the one below that is the best fit for your research. If you are not sure, read the appropriate sections before making your selection.

☒ Life sciences ☐ Behavioural & social sciences ☐ Ecological, evolutionary & environmental sciences

For a reference copy of the document with all sections, see nature.com/documents/nr-reporting-summary-flat.pdf

Life sciences study design

All studies must disclose on these points even when the disclosure is negative.

Sample size	This study includes a total of 8 samples with 6 bone marrow aspirates from patients diagnosed with precursor/overt multiple myeloma (2 monoclonal gammopathy of undetermined significance, 1 smoldering multiple myeloma, 2 newly diagnosed multiple myeloma, 1 relapsed refractory multiple myeloma) and 2 peripheral blood draws with 1 from a patient with plasma cell leukemia and the other from a normal donor.
Data exclusions	All samples were analyzed and the data is provided.
Replication	The single cell data can not be reproduced since it is patient provided time point-specific bone marrow aspirate or peripheral blood draw.
Randomization	This is not relevant to our study. This was an observational investigation of biomarkers associated with disease found using liquid biopsy techniques.
Blinding	This is not relevant to our study. This was an observational investigation of biomarkers associated with disease found using liquid biopsy techniques.

Reporting for specific materials, systems and methods

We require information from authors about some types of materials, experimental systems and methods used in many studies. Here, indicate whether each material, system or method listed is relevant to your study. If you are not sure if a list item applies to your research, read the appropriate section before selecting a response.

Materials & experimental systems

n/a	Involved in the study
☐	☒ Antibodies
☒	☐ Eukaryotic cell lines
☒	☐ Palaeontology and archaeology
☒	☐ Animals and other organisms
☐	☒ Clinical data
☒	☐ Dual use research of concern

Methods

n/a	Involved in the study
☒	☐ ChIP-seq
☒	☐ Flow cytometry
☒	☐ MRI-based neuroimaging

Antibodies

Antibodies used

The slides were then stained with a primary antibody cocktail containing mouse anti-human CD138 (B-A38, MCA2459GA, Bio-Rad, Hercules, CA) and mouse anti-human CD45 Alexa Fluor® 647 monoclonal antibody (F10-89-4, MCA87A647, AbD Serotec, Raleigh, NC), for 1 hour. Antibodies had previously been validated as a part of assay development study for MM [49]. The slides were washed with TBS after primary staining. Thereafter, slides were incubated with goat anti-mouse Alexa Fluor® 555 (A21127, Invitrogen, Carlsbad, CA) and counterstained with 4, 4-diamidino-2-phenylindole (DAPI; D1306, ThermoFisher, Waltham, MA). Slides then underwent imaging mass cytometry staining using CD20 (Rabbit_IgG_SP32, Nd142, Abcam); CD38 (Rabbit_IgG_EPR2269-219 ,Sm152, Abcam); CD81 (Rabbit_IgG_EPR21916, Er167, Abcam); CD4 (Rabbit_IgG_EPR6855, Yb176, Abcam); lambda light chain (Rabbit_IgG_EPR5367-62, Eu151, Abcam); CD31 (Rabbit_IgG_EPR3094, Er168, Abcam); CD28 (Rabbit_IgG_EPR22076, Eu153, Abcam); MUM1 (Rabbit_IgG_EP5699, Gd155, Abcam); BCMA (Rabbit_IgG_EPR22457-260, Nd150, Abcam); BCM (Rabbit_IgG_EPRBOB-R1-F1-24, Nd150, Abcam); c-kit/CD117 (Rabbit_IgG_YR145, Nd145, Abcam); SLAMF7/CS1 (Rabbit_IgG_EPR22948-114, Sm147, Abcam); kappa light chain (Rabbit_IgG_EPR5539-105-4, Tm169, Abcam); Syndecan/ CD138 (Rabbit_IgG_EPR6454, Nd148, Abcam); CD27 (Rabbit_IgG_EPR8569, Nd144, Abcam); PRDM1/ Blimp1 (Rabbit_IgG_EPR16655, Nd146, Abcam); CD63 (Rabbit_IgG_EPR22458-280, Sm154, Abcam); ICAM3 (Rabbit_IgG_EPR3994-123, Gd158, Abcam);BAFF/ CD257(Rabbit_IgG_EPR22238, Gd160, Abcam); CD74 (Rabbit_IgG_EPR4064, Dy161, Abcam); IGF1 receptor/ CD221 (Rabbit_IgG_EPR19322, Dy163, Abcam); BST2/ Tetherin/ CD317 (Rabbit_IgG_EPR20202-169, Dy164, Abcam); Cyclin D1 (Rabbit_IgG_SP4, Er166, Abcam); UBA52 (Rabbit_IgG_EPR4546, Lu175, Abcam); CD56 (Mouse_IgG2b k_NCAM16.2, Sm149, Fluidigm); pS6 (Mouse_IgG1_N7-548, Yb172, Fluidigm);CD3 (Rabbit_IgG_Polyclonal, Er170, Fluidigm); CD44 (Rat_IgG2bk_IM7, Yb171, Fluidigm); CD61 (Mouse_IgG1_VI-PL2, Bi209, Fluidigm); CD45-RO (Mouse_IgG2a_UCHL1, Yb173, Fluidigm); CD45 (Mouse_IgG1k_HI30, Y89, Fluidigm); CD8a (Rabbit_IgG_D8A8Y, Dy162, Fluidigm); HLA-DR (Mouse_IgG2ak_L243, Yb174, Fluidigm); IGLL5 (Rabbit_IgG_polyclonal, Nd143, Thermo Fisher);CD229 (Rabbit_IgG_polyclonal, Tb159, Thermo Fisher); DNA1 (Cell-ID™ Intercalator, Ir191, Fluidigm), and DNA2 (Cell-ID™ Intercalator, Ir193, Fluidigm.)

Validation

Overall performance, including sensitivity and specificity, in both healthy donors and cancer patients was conducted as described previously with references provided in the text.

Clinical data

Policy information about clinical studies

All manuscripts should comply with the ICMJE guidelines for publication of clinical research and a completed CONSORT checklist must be included with all submissions.

Clinical trial registration

Samples collected from patients on the MD Anderson Trial (IRB: UP-19-0033) between 2019-2020.

Study protocol

Full study protocol is available upon request.

Data collection

Data was collected during the recruitment and treatment period and provided as de-identified information in a secure database.

Outcomes

This was an observational study without pre-defined outcome measures.
